# Perseveration by NK1R-/- (‘knockout’) mice is blunted by doses of methylphenidate that affect neither other aspects of their cognitive performance nor the behaviour of wild-type mice in the 5-Choice Continuous Performance Test

**DOI:** 10.1177/0269881116642541

**Published:** 2016-04-19

**Authors:** Katharine Pillidge, Ashley J Porter, Jared W Young, S Clare Stanford

**Affiliations:** 1 Department of Neuroscience, Physiology and Pharmacology, University College London, London, UK; 2 Department of Psychiatry, University of California San Diego, La Jolla, CA, USA; 3 Research Service, VA San Diego Healthcare System, San Diego, CA, USA

**Keywords:** ADHD, false alarms, impulsivity, inattentiveness, NK1 receptor, perseveration, premature responses

## Abstract

The underlying cause(s) of abnormalities expressed by patients with attention deficit hyperactivity disorder (ADHD) have yet to be delineated. One factor that has been associated with increased vulnerability to ADHD is polymorphism(s) of *TACR1*, which is the human equivalent of the rodent NK1 (substance P-preferring) receptor gene (*Nk1r*). We have reported previously that genetically altered mice, lacking functional NK1R (NK1R–/–), express locomotor hyperactivity, which was blunted by the first-line treatment for ADHD, methylphenidate. Here, we compared the effects of this psychostimulant (3, 10 and 30 mg/kg, intraperitoneally) on the behaviour of NK1R-/- mice and their wild types in the 5-Choice Continuous Performance Test, which emulates procedures used to study attention and response control in ADHD patients. Methylphenidate increased total trials (a measure of ‘productivity’) completed by wild types, but not by NK1R-/- mice. Conversely, this drug reduced perseveration by NK1R-/- mice, but not by wild types. Other drug-induced changes in key behaviours were not genotype dependent, especially at the highest dose: for example, % omissions (an index of inattentiveness) was increased, whereas % false alarms and % premature responses (measures of impulsivity) declined in both genotypes, indicating reduced overall response. These findings are discussed in the context of the efficacy of methylphenidate in the treatment of ADHD. Moreover, they lead to several testable proposals. First, methylphenidate does not improve attention in a subgroup of ADHD patients with a functional deficit of TACR1. Second, these patients do not express excessive false alarms when compared with other groups of subjects, but they do express excessive perseveration, which would be ameliorated by methylphenidate.

## Introduction

We have suggested that the abnormal behavioural profile of mice with functional ablation of the neurokinin-1 receptor (*Nk1r*) gene (NK1R-/-) is analogous to that of a subgroup of attention deficit hyperactivity disorder (ADHD) patients with polymorphism(s) of the *TACR1* gene (the human equivalent of *Nk1r*; Sharp et al., 2014; Yan et al., 2010). This proposition is based on our evidence that male NK1R-/- mice express locomotor hyperactivity compared with their wild types (Fisher et al., 2007; Herpfer et al., 2005; Porter et al., 2015a, 2015b), which is diminished by treatment with *d*-amphetamine or methylphenidate (Yan et al., 2009). These mutant mice also display impaired cognitive performance and response control in the 5-Choice Serial Reaction Time Task (5CSRTT). Typically, they score more % premature responses (an index of impulsivity) and % omissions (an index of inattentiveness) than wild types (Dudley et al., 2013; Porter 2015a, 2015b; Yan et al., 2011), especially when tested for the first time (Weir et al., 2014). It is striking that these three behavioural abnormalities meet the diagnostic criteria for ADHD in humans.

All the drugs that are licensed to treat ADHD come from three generic groups (Bolea-Alamañac et al., 2014; Heal et al., 2009). Amphetamine (together with its pro-drug, lisdexamfetamine) and methylphenidate are competitive substrates for noradrenaline/dopamine transporters and are classified as psychostimulants. Guanfacine and clonidine are α_2_-adrenoceptor agonists, while atomoxetine is a selective noradrenaline reuptake inhibitor. Prompted by evidence outlined above, we have compared the effects of drugs from all three classes on the behaviour of NK1R-/- mice and their wild type counterparts. So far, we have reported that the locomotor hyperactivity of NK1R-/- mice was prevented by both *d*-amphetamine and methylphenidate (Yan et al., 2010); their inattentiveness (% omissions) in the 5CSRTT was reduced by a low dose of guanfacine (Pillidge et al., 2014a); and their impulsivity (% premature responses) was reduced by atomoxetine (Pillidge et al., 2014b). Guanfacine also reduced premature responses, but only at a high dose that diminished the incidence of this behaviour in wild types as well (Pillidge et al., 2014a).

Recently, we compared the behaviour of NK1R–/- mice and their wild types in the 5-Choice Continuous Performance Test (5C-CPT; Porter et al., 2016). This procedure is regarded as an analogue of those used to study human cognitive performance because it incorporates both ‘no-go’ (non-target) and ‘go’ (target) light signals, unlike the 5CSRTT, which uses only the latter signal/response contingency. This refinement enables the scoring of both false alarms (response disinhibition) and premature responses (waiting/motoric impulsivity): these are two different aspects of impulsive behaviour (Evenden, 1999), which are mechanistically dissociable (Young et al., 2011). Another advantage of the 5C-CPT is that it not only scores subjects’ total target responses (hit rate), but also enables the evaluation of subjects’ ability to distinguish between target and non-target stimuli (i.e. to respond to ‘go’ but not to ‘no-go’ signals; Sensitivity Index [SI]), as well as their innate bias to respond versus to withhold a response (‘caution’; Responsivity Index [RI]; see Young et al., 2009).

Here, we compared the effects of methylphenidate on attentional performance and response control of NK1R-/- mice and their wild types in the 5C-CPT. Because NK1R-/- mice completed more trials (‘total trials’) than wild types in our previous study using this procedure (Porter et al., 2016), we also compared the effects of methylphenidate on this measure of ‘productivity’ in the two genotypes.

Despite the undisputed benefits of methylphenidate in treating ADHD (Epstein et al., 2014; Faraone et al., 2006; Grizenko et al., 2013; Spencer et al., 2005), approximately 35% of patients do not respond to this drug (Hodgkins et al., 2012). An objective of this study was to establish whether wild type and NK1R-/- mice differ in their behavioural response to methylphenidate in the 5C-CPT. If so, this would suggest that the status of the *TACR1* gene and/or TACR1 receptor could similarly influence the efficacy of this drug treatment in ADHD patients, and that polymorphism(s) of *TACR1* could serve as a biomarker for this subgroup of patients.

## Method

### Apparatus

The apparatus (Med Associates, St. Albans, VT) was controlled by a Smart Ctrl Package 8IN/16OUT and an interface (MED-PC for Windows) with software that had been modified to incorporate ‘no-go’ as well as ‘go’ signals during Stages 3 and 4 of training and the extended test trial of the 5C-CPT (see Young et al., 2009).

### Animals

All experimental procedures complied with the Animals (Scientific Procedures) Act (UK) [2010/63/EU] and received local ethical approval at University College London. The protocol for training mice in the 5C-CPT is explained fully elsewhere (Porter et al., 2016; Young et al., 2009), but essential elements of the procedure are reported below.

Twelve inbred male mice of each genotype were used. These mice express all abnormalities seen in ADHD (locomotor hyperactivity, impulsive behaviour, inattentiveness and perseveration), whereas their excessive impulsivity (but none of their other abnormal behaviours) seems to arise from an interaction between a deficit of functional NK1R and the breeding environment (Porter et al., 2015b). All were six to seven weeks of age at the start of training, and they shared the same genetic background (129/Sv×C57BL/6J, crossed with outbred MF1 mice, many (>10) generations ago; de Felipe et al., 1998). The wild types (weight 30–34 g) were taken from two litters, and NK1R-/- mice (weight 29-31 g) from three litters. Only littermates were group housed in each home cage (two to five per cage). The cages were cleaned twice weekly (bedding; 3Rs Bedding Pty Ltd) and offered environmental enrichment comprising cardboard tunnels and nesting material. The environment was held at 21±2°C, 45±5% humidity and a 12-hour light/dark cycle (increased in steps from 07.00-8.00 hours and reduced in steps from 19.00-20.00 hours). The mice had free access to water, but food (2018 global Rodent Diet, Harlan) was restricted to maintain their body weight at 90% free-feeding baseline. If, at the start of any day, the body weight of a mouse had fallen below 90%, they were allowed some free feeding time that day to restore their body weight.

The animals were fed immediately after training/testing (Monday to Friday). At weekends, they were given half the daily quota in the morning and the remainder in the afternoon (after 16.00 hours).

### Training and testing in the 5C-CPT

Each mouse was assigned to one of four test chambers, which were counterbalanced for genotype, time of day (for training/testing) and litter. Training took place every weekday (described fully in Porter et al., 2016; Young et al., 2009). Half the animals were trained/tested in one of three morning sessions, between 10.00 and 12.00 hours; the remainder were trained/tested in one of three afternoon sessions, between 13.00 and 15.00 hours. Each animal was trained/tested at the same time each day. The criteria for graduation through each of the four stages of training are explained in full in Young et al. (2009) with modifications specified in Porter et al. (2016).

On the first Friday after matching the criteria for graduation in the final stage of training, the performance of treatment-naïve animals was evaluated in a single, extended test session (i.e. no injection: ‘NI-1’). Full details of the test parameters are given in Porter et al. (2016). In brief, the number and duration of the series of trials was 250 trials or 60 minutes, whichever occurred first. The ratio of target to non-target signals was 5:1, and the range of the variable inter-trial intervals (VITIs) was 7-11 seconds. Delivery of all these variables was automated and fully randomised. The performance of the mice during each stage of training and in this NI-1 test are reported in Porter et al. (2016).

### Treatments

Starting one week after this test, the mice were retested once weekly on Fridays, 30 minutes after treatment with either methylphenidate hydrochloride (Sigma Aldrich; 3, 10 or 30 mg/kg, intraperitoneally [i.p.]; 10 mL/kg; ‘MPH3,’ ‘MPH10’ and ‘MPH30’), vehicle (0.9% sterile saline, i.p.: 10 mL/kg; ‘VEH’) or in a second (baseline) test session with no injection (here referred to as ‘NI-2’). Each mouse received each test condition only once, and the sequence for each of the five test conditions was counterbalanced across subjects (defined by a pseudo-Williams’ Latin square). Between test days, the mice were retrained in order to re-establish pretest baseline performance.

The choice of drug doses was informed by a pilot dose-range test, carried out at the end of a previous experiment on a different batch of mice, together with published reports of its effects on locomotor activity and cognition (e.g. Beaulieu et al., 2006; Keck et al., 2013; Siesser et al., 2005; Yan et al., 2010). Detailed pharmacokinetic considerations of what doses are therapeutically equivalent to those in humans cannot be certain. For instance, false alarms in a visual discrimination test of rats were diminished by an oral dose of 0.5 mg/kg methylphenidate (Berridge et al., 2006), but 2 mg/kg i.p. in rats was optimal for improvement in a working memory test of cognition (Spencer et al., 2015). The latter finding suggests that the range of doses used in this study was appropriate for detecting changes in cognition, given that higher doses would normally be needed in mice than in rats, but lower oral doses than those given i.p. This inference is also consistent with a report that oral administration of 3 mg/kg racemic methylphenidate to B6C3F1 mice produced a plasma concentration (*C*_max_) within the therapeutic range in ADHD patients (6–40 ng/mL; Manjanatha et al., 2008). A further factor is that compared with oral administration, i.p. injection of methylphenidate causes a more prolonged plasma elimination time and locomotor response, which is arguably more therapeutically relevant (Gerasimov et al., 2000).

The behavioural data were captured and stored online. One wild-type mouse was withdrawn from this study for reasons unrelated to the procedure. No data from this animal were included in the analysis.

### Performance variables and statistical analysis

As in the 5CSRTT, we scored the following aspects of the animals’ performance: % accuracy, % omissions, % premature responses and perseverative responses, latency to correct response and latency to collect the reward. We further calculated the proportion of false alarms (an index of response disinhibition), the latency to false alarms; the hit rate (animals’ total responses to target trials), RI (an index of an animal’s biased tendency to respond to a signal, whether appropriate or not) and SI (an index of an animal’s attentional performance responding to the target versus non-target signals). Details of these calculations are specified in Young et al. (2009, 2011, 2013b).

InVivoStat (v2.3.0.0; Clark et al., 2012) was used to analyse the data. Diagnostic plots were constructed routinely to check for normality of the data-set and equality of the variance of the samples. When necessary, the data were √(score)-, Log_10_(score+1)-, or rank-transformed to optimise the homogeneity of variance across the experimental groups before proceeding with subsequent parametric statistical analyses. Mead’s resource equation was used to confirm that sample sizes were adequate to detect statistical significance, which was set at *p*<0.05.

Statistical analysis of the data used mixed model analysis of variance. We first compared all five experimental groups (NI-2, VEH, MPH3, MPH10 and MPH30) to look for overall differences in the main (between subjects) factors, genotype and time of day, and the within-subjects factor, drug. Unlike behaviour during NI-1 (reported in Porter et al., 2016), there were neither main effects of time of day nor any interactions between time of day and other factors of interest, and so the data for all experimental groups were collapsed across this factor. Comparison of NI-2 and VEH was carried out to look for changes arising from the stress of the i.p. injection, which is often regarded as a control procedure (but see Stanford, 1996; Stanford et al., 1984). The effects of methylphenidate (drug and drug×genotype) are based on comparisons with those of the vehicle injection, but comparisons with uninjected mice (NI-2) were carried out when appropriate. The post hoc least significant difference test was used to compare paired groups of data.

We also used the number of perseverative responses by individual mice as the covariate in an analysis of covariance of latency to collect the reward in order to establish whether a genotype difference in perseveration could account for any group differences in this measure.

## Results

### Methylphenidate reduced impulsive behaviours by NK1R-/- and wild-type mice

**Figure 1.**
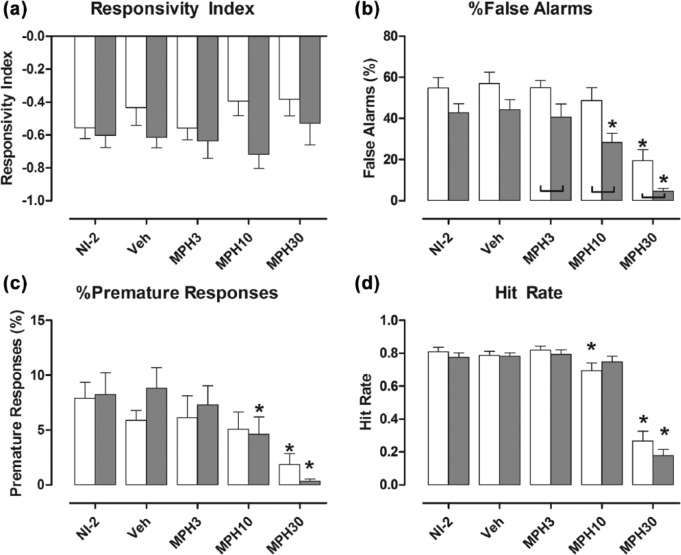
Methylphenidate did not affect the Responsivity Index of the two genotypes (a), but the highest dose (30 mg/kg) reduced % false alarms (b), % premature responses (c) and the hit rate (d) by both genotypes. Bars show mean±standard error of the mean (SEM) score for the five test conditions. White bars: wild types; shaded bars: NK1R-/- mice; NI-2: no-injection (results for NI-1 are reported in Porter et al., 2016); VEH: vehicle; MPH3, MPH10 and MPH30: methylphenidate 3, 10 and 30 mg/kg, respectively. Lines linking pairs of data indicate a statistically significant difference between the two genotypes at *p*<0.05 at least. *p<0.05, by comparison with vehicle injection test condition. Details of exact *p-*values are given in the text. *N*=11 or 12 per group.

Methylphenidate had no overall effect on the RI of the two genotypes (*F*[1, 21]=3.18; *p*=0.089; Figure 1(a)); that is, it did not affect the tendency of the two genotypes to respond to either light signal.

NK1R-/- mice carried out proportionately fewer false alarms than wild types overall (*F*[1, 21]=9.15; *p*=0.006]. The incidence (%) of false alarms by NK1R-/- mice was lower than in wild types after treatment with any dose of methylphenidate. The intermediate drug dose of methylphenidate (10 mg/kg) reduced % false alarms by NK1R-/- mice only (VEH vs. MPH10: *p*=0.005), but the highest dose reduced this behaviour in both genotypes (*F*[3, 63]=41.11; *p*<0.001: VEH vs. MPH30; WT: *p*<0.001; KO: *p*<0.001; Figure 1(b)). However, there was no interaction between drug treatment and genotype.

Methylphenidate reduced premature responses in both genotypes overall (*F*[3, 63]=18.95; *p*<0.001), but unlike % false alarms, there was no genotype difference in the incidence of premature responses after any dose of drug. Compared with vehicle, the intermediate dose (10 mg/kg) reduced % premature responses in NK1R–/– mice only (VEH vs. MPH10: *p*=0.021]. However, the highest dose (30 mg/kg) reduced % premature responses in both genotypes (VEH vs. MPH30, WT: *p*<0.001; KO: *p*<0.001; Figure 1(c)). Again, there was no interaction between drug treatment and genotype.

The intermediate dose of methylphenidate (10 mg/kg) reduced the hit rate by wild types only, but the highest dose reduced this behaviour in both genotypes (VEH vs. MPH30: *F*[3, 60]=53.73; *p*<0.001; Figure 1(d)). However, there was no interaction between drug treatment and genotype.

### Attention and vigilance, but not accuracy, were reduced by methylphenidate

**Figure 2.**
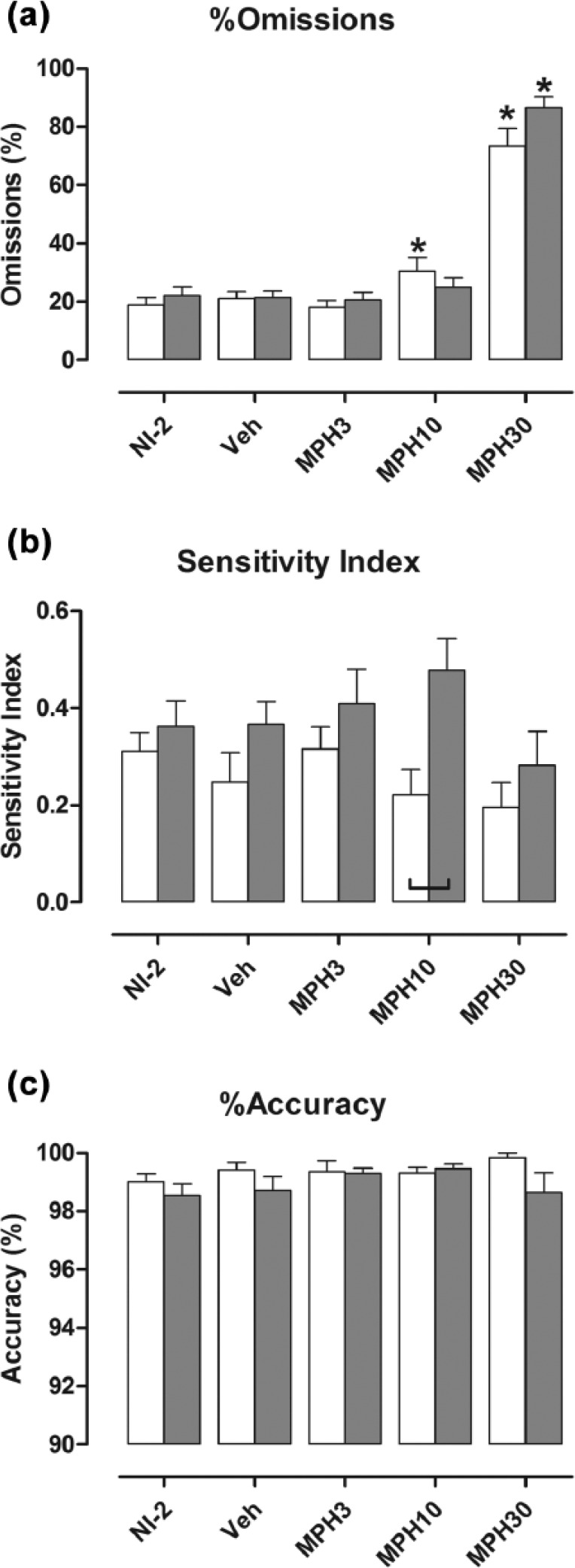
% omissions (a), Sensitivity Index (b) and % accuracy (c) did not differ in the two genotypes during NI-2. Bars show mean±SEM score for the five test conditions. White bars: wild types; shaded bars: NK1R-/- mice; NI-2: no-injection (results for NI-1 are reported in Porter et al., 2016); VEH: vehicle; MPH3, MPH10 and MPH30: methylphenidate 3, 10 and 30 mg/kg, respectively. The highest dose of methylphenidate increased % omissions in both genotypes, but caused an overall reduction in the Sensitivity Index (b) and did not affect accuracy (c). Lines linking pairs of data indicate a statistically significant difference between the two genotypes at *p*<0.05 at least. *p<0.05, by comparison with vehicle injection test condition. Details of exact *p*-values are given in the text. *N*=11 or 12 per group.

Methylphenidate increased % omissions in both genotypes overall (*F*[3, 63]=143.19; *p*<0.001; Figure 2(a)), but there was no genotype difference in this measure of inattentiveness after any drug dose. After treatment with the intermediate dose (10 mg/kg), a small increase was evident in wild types (VEH vs. MPH10: *p*=0.026), but not NK1R–/– mice (VEH vs. MPH10: *p*=0.402). The highest dose (30 mg/kg) increased % omissions in both genotypes (VEH vs. MPH30, WT: *p*<0.001; KO: *p*<0.001]. Nevertheless, there was no interaction between drug treatment and genotype.

An overall genotype difference in the SI suggested that NK1R-/– mice were more vigilant than wild types (*F*[1, 21]=5.02; WT vs. KO: *p*=0.036), especially after treatment with the intermediate dose of methylphenidate (10 mg/kg; Figure 2(b)) However, methylphenidate reduced the SI overall (*F*[3, 60]=3.57; *p*=0.019), and there was no interaction between drug treatment and genotype. Finally, methylphenidate did not affect % accuracy of either genotype (Figure 2(c)).

### Perseveration by NK1R-/- mice, but not wild types, is prevented by methylphenidate

**Figure 3.**
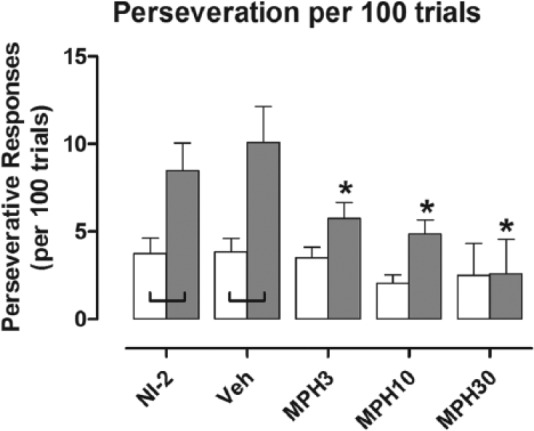
Perseveration by NK1R–/– mice was higher than for wild types. All doses of methylphenidate reduced this behaviour in NK1R-/- mice but not wild types. Bars show mean±SEM score for the five test conditions. White bars: wild types; shaded bars: NK1R-/- mice; NI-2: no-injection (results for NI-1 are reported in Porter et al., 2016); VEH: vehicle; MPH3, MPH10 and MPH30: methylphenidate 3, 10 and 30 mg/kg, respectively. Lines linking pairs of data indicate a statistically significant difference between the two genotypes at *p*<0.05 at least. *p<0.05, by comparison with vehicle injection test condition. Details of exact *p*-values are given in the text. *N*=11 or 12 per group.

Perseveration by NK1R–/– mice was higher than that of wild types overall (*F*[1, 21]=8.31; *p*=0.009), especially during NI-2 (*p*=0.008) and after vehicle injection (*p*=0.001; Figure 3). All doses of methylphenidate blunted this behaviour in NK1R-/- mice (*F*[3, 63]=11.29; *p*<0.001; VEH vs. MPH3: *p*=0.017; MPH10: *p*=0.003; MPH30: *p*<0.001), but none affected the wild types, most likely because of a floor effect. Nonetheless, an interaction between the drug treatment and genotype just missed the criterion for statistical significance (genotype×drug: *F*[3, 63]=2.71; *p*=0.053).

### The effect of methylphenidate on response latencies differed in the two genotypes

**Figure 4.**
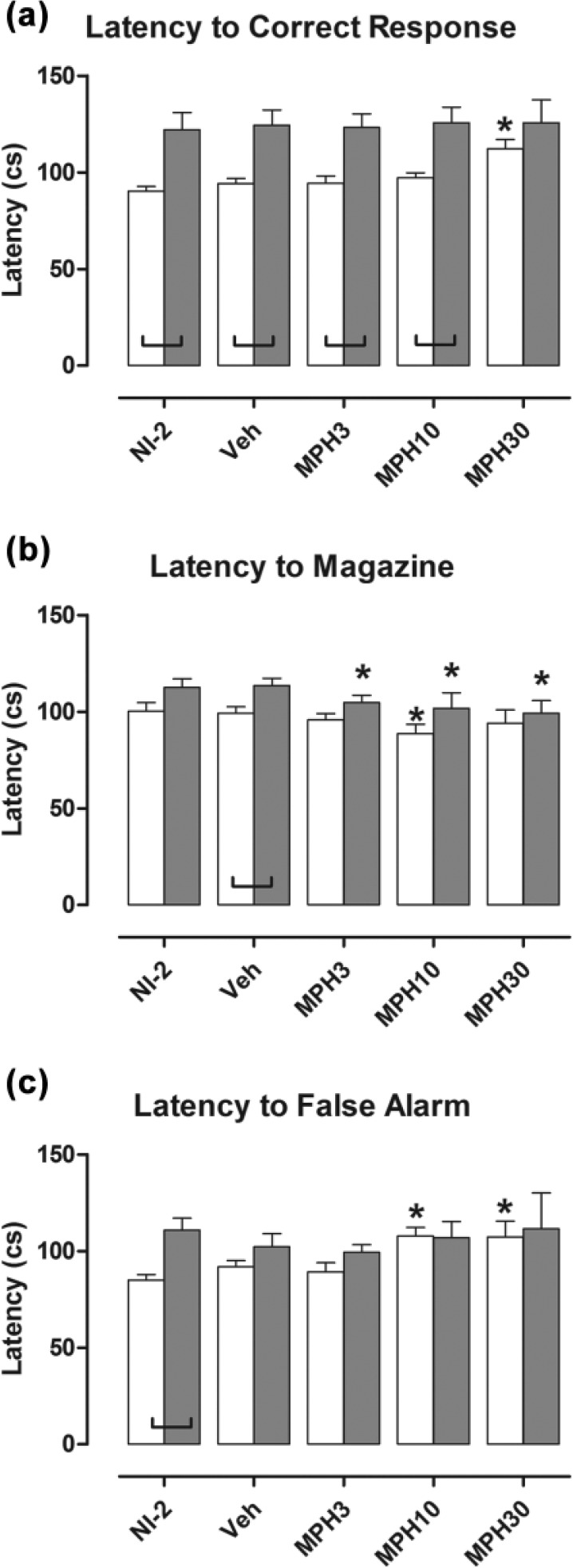
Latency to correct response (a) was higher in NK1R–/– mice than in wild types. The highest dose of methylphenidate (MPH30) increased this measure in wild types only. Methylphenidate reduced the latency to collect the reward (b), especially in NK1R-/- mice, but only the intermediate dose affected both genotypes. The intermediate and highest dose of methylphenidate increased the latency to false alarms (c) for wild types only. Bars show mean±SEM score for the five test conditions. White bars: wild types; shaded bars: NK1R-/- mice; NI-2: no-injection (results for NI-1 are reported in Porter et al., 2016); VEH: vehicle; MPH3, MPH10 and MPH30: methylphenidate 3, 10 and 30 mg/kg, respectively. Lines linking pairs of data indicate a statistically significant difference between the two genoptypes at *p*<0.05 at least. *p<0.05, by comparison with vehicle injection test condition. Details of exact *p*-values are given in the text. *N*=11 or 12 per group.

The latency to correct response was longer in NK1R–/– mice than in wild types overall (*F*[1, 21]=20.29; *p*<0.001; Figure 4(a)). The highest dose of methylphenidate increased this latency in wild types but not in NK1R-/- mice (genotype×drug: *F*[3, 60]=4.84, *p*=0.004; VEH vs. MPH30, WT: *p*<0.001; KO: *p*=0.461). As a consequence, the genotype difference in this performance measure was abolished by the highest dose of drug.

By contrast, methylphenidate reduced the latency to collect the reward overall (*F*[3, 60]=7.92; *p*<0.001], especially in NK1R-/- mice (Figure 4(b)). Whereas the 10 mg/kg dose reduced this latency in both genotypes (VEH vs. MPH10, WT: *p*=0.002; KO: *p*<0.001), the lower (3 mg/kg) and higher (30 mg/kg) doses reduced this measure for NK1R-/– mice only (VEH vs. MPH3: *p*=0.040; MPH30: *p*<0.001). Nevertheless, there was no interaction between drug treatment and genotype. The genotype difference in the latency to collect the reward was not evident when perseveration was treated as a covariate in the statistical analysis (*F*[1, 21]=1.03; *p*=0.322]; that is, the delay incurred by perseveration accounted for the longer latency of NK1R–/- mice. However, methylphenidate still caused an overall reduction in this measure (*F*[3, 59]=4.24; *p*=0.009).

Latency to false alarms was higher in NK1R–/– mice than wild types overall (*F*[1, 21]=11.33; *p*=0.003; Figure 4(c)). Methylphenidate had different effects on the two genotypes (genotype×drug: *F*[3, 56]=2.75; *p*=0.050). Whereas there was an increase when wild types were given either 10 mg/kg or 30 mg/kg (VEH vs. MPH10: *p*=0.050; MPH30: *p*=0.048), this was not the case for NK1R-/- mice.

### Methylphenidate differentially affected, by dose, total trials completed by NK1R-/- mice and wild types

**Figure 5.**
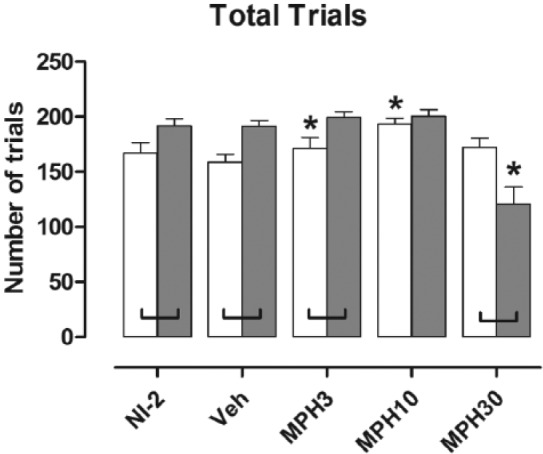
Total trials completed by NK1R-/- mice during the extended test sessions of the 5C-CPT. Bars show mean±SEM score for the five test conditions. White bars: wild types; shaded bars: NK1R-/- mice; NI-2: no-injection (results for NI-1 are reported in Porter et al., 2016); VEH: vehicle; MPH3, MPH10 and MPH30: methylphenidate 3, 10 and 30 mg/kg, respectively. Lines linking pairs of data indicate a statistically significant difference between the two genotypes at *p*<0.05 at least. *p<0.05, by comparison with vehicle injection test condition. Details of exact *p*-values are given in the text. Treatment with 3 or 10 mg/kg methylphenidate (i.p.) increased the number of total trials completed by wild types, whereas the highest dose (30 mg/kg, MPH30) reduced total trials completed by NK1R-/- mice. *N*=11 or 12 per group.

Total trials completed was higher for NK1R–/– mice than it was for wild types overall for the series of five test conditions (*F*[1, 21]=4.61; *p*=0.044; Figure 5). Methylphenidate affected total trials in a genotype-dependent manner (genotype×drug: *F*[3, 63]=12.58; *p*<0.001). Compared with vehicle-treated mice, both 3 mg/kg and 10 mg/kg *increased* (7.5% and 21.4%, respectively) total trials completed by wild types, but not those completed by NK1R-/- mice (VEH vs. MPH3: *p*=0.049; MPH10: *p*<0.001). By contrast, the highest drug dose (30 mg/kg) *reduced* (-36.7% cf. vehicle) this measure for NK1R-/- mice but not for wild types (VEH vs. MPH30: *p*<0.001). At this dose, the score for wild types was higher than that for NK1R-/- mice (*F*[3, 63]=10.11; *p*<0.001).

## Discussion

We have proposed that behavioural abnormalities of NK1R-/- mice resemble those of ADHD patients who have functional disruption of the *TACR1* gene (the human equivalent of *Nk1r*; Sharp et al., 2014; Yan et al., 2010, 2011). Although the locus of the *TACR1* polymorphism and its functional consequences have yet to be defined, this proposal could be important in light of confirmed associations between *TACR1* polymorphisms, ADHD and other co-morbidities. For instance, there is evidence for an association between alcohol misuse and *TACR1* polymorphism(s) (see Blaine et al., 2013), which could help explain why ADHD is a risk factor for co-morbid alcohol misuse (Wilens et al., 2011). Similarly, there is confirmed association between both *TACR1* polymorphism(s) (Sklar et al., 2008; see Sharp et al., 2014) and ADHD (Torres et al., 2015) with bipolar disorder.

In a previous study, the locomotor activity of NK1R-/- mice in a light/dark exploration box was blunted by the psychostimulant, methylphenidate, which is a first-line treatment for ADHD (Yan et al., 2010). Here, we investigated whether methylphenidate reduced inattentiveness and impulsive behaviour (premature responses and false alarms) by NK1R-/- mice in the 5C-CPT. This test emulates procedures used to evaluate attentional performance and response control in humans, including ADHD patients.

The first point to note is that neither inattentiveness nor the impulsive behaviour of the two genotypes differed when they experienced the baseline (treatment-free NI-2) test. In the 5CSRTT, NK1R-/- mice are typically more inattentive and impulsive than wild types when tested for the first time, but these differences dissipate with repeated experience of the test (Weir et al., 2014). However, in the 5C-CPT, neither inattentiveness nor impulsivity of the mice differed, even when they were tested for the first time (see Porter et al., 2016). We have suggested that this is because, in the 5C-CPT, the mice experience the VITI during the latter stages of training, as well as during the test phase. As a consequence (unlike the 5CSRTT), they become familiar with the VITI schedule and expect the latency of the light cue to be unpredictable. If so, this points to unexpected uncertainty in respect of the latency of the light signal as a key factor for detecting behavioural deficits in NK1R-/- mice (Porter et al., 2016). Another could be that the range of latencies of the VITIs in this 5C-CPT test differed from those used in the 5CSRTT, which could give an important clue to the optimal ITI for distinguishing behavioural deficits in the NK1R-/- genotype.

Despite the lack of any differences in attentiveness or impulsive behaviour, there were interesting differences in the effect of methylphenidate on the behaviour of the two genotypes. The highest dose of methylphenidate reduced premature responses and false alarms in both genotypes. However, the hit rate was also reduced and % omissions were increased, suggesting that this dose of methylphenidate caused a non-specific blunting of motor responses. By contrast, the intermediate dose of methylphenidate (10 mg/kg) reduced both premature responses and false alarms by NK1R-/- mice only, without affecting the number of target response trials, indicative of a selective effect in these mice. Although additional test groups are needed to confirm whether NK1R-/- mice are more sensitive to methylphenidate than wild types, it is clear that this response to methylphenidate does not require NK1R and, in fact, a lack of NK1R may mediate heightened sensitivity to methylphenidate. Therefore, importantly, given the lack of a concurrent reduction in hit rate and the lack of an increase in the latency to correct response, latency to collect the reward, latency to false alarms or % omissions, this dose of methylphenidate seems to have a specific effect on impulsive aspects of behaviour in NK1R–/– mice.

About 70% of ADHD patients show improved response control, over a wide range of tests, after treatment with methylphenidate (reviewed by Pietrzak et al., 2006). However, there are many reports that methylphenidate does not reduce ‘commission errors’ (i.e. false alarms plus premature responses) by ADHD patients in CPTs (Slama et al., 2015), except at doses that disrupt other behaviours (Sunohara et al., 1999). This response profile reflects that of wild types after drug treatment in this study. Conversely, there are several reports that a low dose of methylphenidate reduces commission errors without affecting omission errors (Aggarwal and Lillystone, 2000; Aman et al., 1984; Bron et al., 2014; O’Toole et al., 1997; reviewed by Riccio et al., 2001), which matches the response profile for NK1R-/- mice. Collectively, these findings lead to the prediction that impulsive behaviour of ADHD patients with *TACR1* polymorphism(s), but not that of other subjects, might be diminished by a dose of methylphenidate that does not impair their attention.

We are aware of only one other study of the effects of methylphenidate on the behaviour of rodents in the 5C-CPT (Tomlinson et al., 2014). An important finding was that methylphenidate reduced the impulsivity of high-impulsive rats (for an equivalent observation in humans, see Aman et al., 1984), but increased it in low-impulsive rats. Nevertheless, it is unlikely that individual differences in baseline performance in the present study constrained the effects of methylphenidate on false alarms at least. This interpretation is based on our evidence that the incidence of false alarms tended to be higher in wild types, at baseline, and yet the intermediate dose of methylphenidate blunted this behaviour only in NK1R-/- mice.

Regarding measures of attention, the low and intermediate doses of methylphenidate did not reduce baseline % omissions by either genotype, possibly because of a floor effect (see below). Some human studies have noted an improvement in attention after treatment with this drug (Bédard et al., 2015; Rubia et al., 2009; Schachar et al., 2008). Presumably, these are subjects with high inattentiveness at baseline (see also Tomlinson et al., 2014). However, our findings are consistent with reports that methylphenidate does not improve attention of ADHD patients in CPTs (e.g. Aggarwal et al., 2000; O’Toole et al., 1997; Slama et al., 2015). The increase in % omissions, in both genotypes, following treatment with the highest drug dose evidently does not depend on activation of, or a lack of, NK1R.

The low % omissions of NK1R-/- mice in this study contrasts with our findings from studies using the 5CSRTT, in which this genotype typically expressed excessive omission errors, when tested with a prolonged but fixed inter-trial interval (‘LITI’; Dudley et al., 2013; Porter et al., 2015a; Yan et al., 2011). We have not carried out a randomised, head-to-head comparison of the two genotypes in the 5CSRTT and 5C-CPT. However, our findings so far suggest that the longer LITI, used in the 5CSRTT, is more effective than the protocol in this 5C-CPT for discerning a higher proportion of omission errors in NK1R–/– mice than in wild types (Porter et al., 2015a; Weir et al., 2014; Yan et al., 2011; see also Porter et al., 2016).

None of the doses of methylphenidate improved % accuracy, an index of selective attention, in this 5C-CPT. It is evident that the effects of methylphenidate on different aspects of attention in this 5C-CPT (% accuracy and % omissions) are strongly dissociated. Although the lack of any response to methylphenidate could be explained by the high baseline accuracy of untreated mice (close to 100% at baseline), there are reports that methylphenidate has no effect on the % accuracy of rats in the 5CSRTT, despite their high incidence of incorrect responses at baseline (Paterson et al., 2011). However, methylphenidate can increase accuracy in human CPTs (Heiser et al., 2004; Klorman et al., 1991), especially when the task is made more challenging (Coons et al., 1981). In that context, it is interesting that NK1R-/- mice were less accurate than wild types at the onset of Stage 1 of training in the 5C-CPT (Porter et al., 2016), suggesting that habituation to the task eliminates any genotype difference in this measure.

The only behaviour to show a marked genotype difference in NI-2 was perseveration, an abnormality that is also prominent in both NI-1 of this test (Porter et al., 2016) and the 5CSRTT (Dudley et al., 2013; Weir et al., 2014; Yan et al., 2011; Porter et al., 2015a; but see Porter et al, 2015b). Evidently, a deficit in functional NK1R can exacerbate this behaviour. All doses of methylphenidate attenuated perseveration by NK1R–/– mice (even the lowest dose, which did not affect any other behaviour), but none affected wild types, possibly because of a floor effect, a pattern that is consistent with impulsivity-relevant behaviours in the study by Tomlinson et al. (2014). It follows that although a lack of functional NK1R exacerbates perseveration, its reduction, following drug treatment, does not involve activation of NK1R. It is interesting that in the 5CSRTT, *d*-amphetamine similarly reduced perseveration by NK1R–/– mice but not by wild types (Yan et al., 2011), suggesting that the blunting of this behaviour by these two drugs might share a common mechanism. Given that they are both psychostimulants, an obvious primary candidate is an increase in neurotransmission from noradrenergic and/or dopaminergic neurones.

This methylphenidate-induced reduction in perseveration is striking because most compulsive behaviours in ADHD patients are exacerbated by psychostimulants (e.g. Borcherding et al., 1990; Graham and Coghill, 2008; but see Tannock and Schachar, 1992). However, perseveration, in the form of ‘compulsive checking’ in ADHD patients, might be an exception (Gürkan et al., 2010). This possibility that perseveration by mice in the 5C-CPT and 5CSRTT is analogous to this ‘checking’ behaviour in ADHD merits further investigation using a recently developed ‘checking task’ for rodents (Eagle et al., 2014).

The neurobiological explanation for this form of perseveration and its prevention by methylphenidate is unknown. However, there are many reports that perseveration is exacerbated by lesions of mesocorticolimbic dopaminergic neurones (Pioli et al., 2008; Schwabe et al., 2004). The DRD2/DRD3 receptor agonist, quinpirole, which reduces extracellular dopamine in the nucleus accumbens at least (Escobar et al., 2015), provokes a similar response (e.g. Alkhatib et al., 2013). Many reports indicate that a lack of functional NK1R can diminish dopaminergic transmission in the brain (Anderson et al., 1994; Brimblecombe and Cragg, 2015; Kombian et al., 2003; Loonam et al., 2003; Zocchi et al., 2003) especially during heightened arousal (Hutson et al., 2004; Renoldi and Invernizzi, 2006; Yan et al., 2011). It is possible that amelioration of this deficit by methylphenidate, which is a dopamine reuptake inhibitor, helps to prevent perseveration by these mice.

A final finding was that total trials completed by NK1R-/- mice was consistently higher than that by wild types, as in NI-1 (Porter et al., 2016), suggesting that a lack of functional NK1R increases this measure of ‘productivity’. The highest dose of methylphenidate diminished total trials, especially in NK1R-/- mice. However, the substantial fall in the hit rate in both genotypes at this dose points to a non-specific impairment of performance, which does not involve NK1R. In fact, functional NK1R in wild types might even have ameliorated the response to this drug.

The lower doses of methylphenidate (3 and 10 mg/kg) slightly increased total trials completed by wild types, but did not affect NK1R-/- mice. This genotype difference was not due to a ceiling effect for NK1R-/- mice. It is also unlikely to be explained by a drug-induced increase in the motivation, or the ability, of wild types to perform the task because neither their latency to correct response nor latency to false alarms was reduced. We can also exclude a non-specific increase in the animals’ response to the light stimuli because the hit rate and % false alarms for wild types was reduced, rather than increased, by this drug. Overall, the increase in total trials by wild types, induced by low doses of methylphenidate, seems to be explained by enhancement of another as yet unidentified aspect of their engagement with the task that requires functional NK1R.

To the best of our knowledge, the effect of methylphenidate on the productivity of humans in a CPT has not been reported. This remains unknown because human CPTs have a set number of trials that are computer initiated whereas, in the 5C-CPT, all trials are initiated by the mice. Nevertheless, our findings lead us to propose that there could be interesting differences between ADHD patients with polymorphism(s) of *TACR1* and other groups of subjects, both at baseline and after treatment with methylphenidate. Assessment of the performance of patients and mice, along with the effects of methylphenidate, in cross-species, effort-related tasks (Horan et al., 2015; Orsini et al., 2015; Young and Markou, 2015) could provide further support for our premise that although a deficit of functional TACR1/NK1R apparently improves productivity at baseline, these receptors are needed to facilitate the increase in productivity induced by methylphenidate.

## Conclusions

Each aspect of the cognitive performance and response control of wild type and NK1R-/- mice in the 5C-CPT is affected by methylphenidate in a different way. However, the role of NK1R in the response to methylphenidate had no bearing on the influence of these receptors on baseline behaviour. This disparate drug/response profile could help to explain why hitherto it has not been possible to predict whether individual ADHD patients will respond to treatment with methylphenidate (e.g. DuPaul et al., 1994; Elliott et al., 2014; Johnston et al., 2015; Kim et al., 2015). The findings from this study lead us to predict that a subgroup of ADHD patients, with a deficit in functional TACR1, will be more productive (i.e. complete more trials) and express a higher incidence of perseveration than other patients do and that these differences would be diminished by methylphenidate. Moreover, this drug might also reduce false alarms and premature responses by this group of patients at doses that do not disrupt other aspects of their cognitive and motor performance. Identifying and testing ADHD patients with a deficit in functional TACR1 gene and being treated with methylphenidate in the reverse-translated human 5C-CPT (Van Enkhuizen et al., 2013; Young et al., 2013a) would enable these hypotheses to be interrogated.
